# Isolation and Characterization of Neural Progenitor Cells From Bone Marrow in Cell Replacement Therapy of Brain Injury

**DOI:** 10.3389/fncel.2020.00049

**Published:** 2020-03-12

**Authors:** Wen-fang Bai, Yuling Zhang, Weicheng Xu, Weikun Li, Meihui Li, Fengying Yuan, Xun Luo, Mingsheng Zhang

**Affiliations:** ^1^Department of Rehabilitation Medicine, Guangdong Provincial People’s Hospital, Guangdong Academy of Medical Sciences, Guangdong Provincial Institute of Geriatrics, Guangzhou, China; ^2^School of Medical Instrument and Food Engineering, The University of Shanghai for Science and Technology, Shanghai, China; ^3^Stroke Biological Recovery Laboratory, Spaulding Rehabilitation Hospital, a Teaching Affiliate of Harvard Medical School, Charlestown, MA, United States; ^4^Department of Rehabilitation Medicine, Zengcheng District People’s Hospital of Guangzhou, Guangzhou, China; ^5^Department of Rehabilitation Medicine, The First Affiliated Hospital of Guangdong Pharmaceutical University, Guangzhou, China; ^6^Department of Rehabilitation Medicine, Guangdong Provincial Hospital of Integrated Traditional Chinese and Western Medicine, Foshan, China; ^7^Kerry Rehabilitation Medicine Research Institute, Shenzhen, China; ^8^Shenzhen Sanming Project Group, Spaulding Rehabilitation Hospital, a Teaching Affiliate of Harvard Medical School, Charlestown, MA, United States

**Keywords:** mesenchymal stem cells, bone marrow-derived neural progenitor cells, nerve cells, differentiation, cell transplantation

## Abstract

Many studies supported that bone marrow mesenchymal stem cells (BM-MSCs) can differentiate into neural cells, but few researchers detected mature and function of nerve cells, especially *in vivo* study. Some researchers even suggested that BM-MSCs transplantation would not be able to differentiate into functional neural cells. To figure out the dispute, this study examined bone marrow-derived sphere-like cells, harvested *via* neural stem cell suspension culture, then identified as bone marrow-derived neural progenitor cells (BM-NPCs) by finding the expression of neural progenitor cells genes and proteins, neural progenitor cells characteristic and nerve cell differentiation induced through both methods. Moreover, BM-NPCs transplantation showed long-term survival and improved the ethological and histological indexes of brain injury rats, demonstrating functional nervous cells differentiated from BM-NPCs. These *in vitro* and *in vivo* results confirmed BM-NPCs differentiating into mature and functional nerve cells. This study provided valuable experimental data for BM-NPCs, suggesting a potential alternative treatment of central nervous injury disease.

## Introduction

Research on stem cells and regenerative medicine is the most popular in current life science frontiers (Chen et al., 2019; Dupont et al., 2019; Stoddard-Bennett and Reijo Pera, 2019; Yamada et al., 2019). Differentiating into functional neural cells plays an important role in the neural network plasticity after brain injury. The stem cell transplant to treat central nervous injury has made great progress (Xu et al., 2017; Hosseini et al., 2018; Ludwig et al., 2018; Zamproni et al., 2019).

Mesenchymal stem cells (MSCs) are the most studied potential stem cells (Bhartiya, 2019). Global clinical trials on MSCs have been over 750 projects^1^. The United States approved more than 40 items in brain injury in Clinical trials. MSCs will be promising treatment strategies for the recovery of brain damage (Sherman et al., 2019). One of the reasons is that MSCs with strong proliferation and multi-directional differentiation potential can transdifferentiate into neurons and glial cells of ectoderm cells *in vivo* or *in vitro* in an appropriate environment, and plays a vital role of nerve repair (Kabos et al., 2002; Zhang et al., 2004; Munoz et al., 2005; Robinson et al., 2011; Tang et al., 2012; Huat et al., 2015). However, there are some problems in the transplantation of MSCs, such as lack of long-term survival in intracranial and limited direct evidence of nerve regeneration (Matsuse et al., 2011).

Although in recent years, lots of studies supported that bone-marrow MSCs (BM-MSCs) could transdifferentiate into neural cells, *in vitro* most of them (Long et al., 2005; Lei et al., 2007; Sun et al., 2007; Mu et al., 2018; Luo et al., 2019; Ruan et al., 2019), but few researchers could detect mature and function nerve cells, especially *in vivo* study (Tomita et al., 2006; Raedt et al., 2007; Nojiri et al., 2008). Even some researchers suggested that transplanted BM-MSCs were not able to differentiate into functional neural cells, at least expressed a limited set of neural markers and no cells replaced effect (Raedt et al., 2007). But in most cases of BM-MSCs transplantation, functional recovery was recognized even if just a few transplanted cells survived in the host tissue (Parr et al., 2008). The main role of promoting neural functional recovery probably was raised by inhibiting apoptosis, regulating the body’s immune response to reduce inflammation, and so on (Shi et al., 2018).

It is much more than that. The possibility of committed tissue-specific stem cells pre-existing in the bone marrow has not been dealt with adequately. Any trans-differentiation studies employing populations of bone marrow cells should rule out the possibility that the apparently pure hematopoietic stem cell population could, in fact, contain pre-existing tissue-specific stem/progenitors (Kucia et al., 2004). It is reported that mRNA of several early markers for neural is detectable in peripheral blood mononuclear cells (Kucia et al., 2004). Our previous study examined the nerve cells culture environment, including which bone marrow-derived nerve cells may exist a phase of bone marrow-derived neural progenitor cells (BM-NPCs). BM-NPCs might be more suitable than BM-MSCs, served as seed cells for cell transplantation, playing the role of cell replacement therapy in the central nervous injury disease (Bai et al., 2013). Therefore, how to isolate neural progenitor cells from BM-MSCs and directly differentiate these progenitor cells into functional neural cells, looking the convincing proof for BM-NPCs, and observing the bone marrow derived neurons in long-term intracranial survival, and participating in nerve regeneration, are the urgent problems to be solved in clinical cell transplantation for treating brain injury. here, our study provide evidence that a neural progenitor cell population (BM-NPCs) could be separated from BM-MSCs and these BM-NPCs are able to further differentiate into neural cells *in vitro* based on the cell morphology and cell marker expression, and improve damaged brain function after cell transplantation. These results provide valuable experimental data for BM-NPCs in the central nerve regeneration application.

## Materials and Methods

### Isolation and Culture of BM-MSCs

Adult (3 weeks) specific-pathogen-free (SPF)-class SD rats were purchased from the Laboratory Animal Centre of Sun Yat-sen University. Rats BM-MSCs were generated using the whole bone marrow adherent culture method. Briefly, bone marrow was obtained as in our previous study (Bai et al., 2013) and then centrifuged at 1,500 rpm for 5 min. The supernatant was discarded, and the cell pellet was re-suspended in α-MEM medium plus with 10% FBS, transferred into a petri dish, and cultured in an incubator at 37°C and 5% CO_2_. The medium was replaced every 2 days, as the cells were subcultured when the cell confluency reaches 90%.

### Isolation and Culture of BM-NPCs

After two generations of BM-MSCs, cells were detached by trypsin-EDTA and cultured in a serum-free medium of neural stem cells culture medium Neurobal-A with 1% N2-supplement, 2 mmol/L L-glutamine and 20 ng/ml b-FGF and EGF in suspension culture bottles induction. After 48 h, there were cells in suspended growth, using Accutase™ enzyme digestion batches, some of these cells have the ability of proliferation as a sphere suspension growth.

### Flow Cytometry Analysis of BM-MSCs and BM-NPCs

BM-MSCs or BM-NPCs were harvested with trypsin and washed twice with PBS. After filtering through a 200-mesh screen, the cell density was adjusted to 2–6 × 10^6^/ml. The surface markers molecules on the BM-MSCs were then examined by flow cytometry with the following antibodies: CD3-PE, CD4-FITC, CD11b-PE, CD29-FITC, CD34-APC, CD14-APC, CD45-FITC, CD105-APC, and CD133-PE.

### Immunofluorescent Staining

Cells were plated on the coverslips on the six-well plates. After attaching to the plates, the cells were fixed with 4% Paraformaldehyde (PFA) for 30 min, and then washed with PBS, permeabilized with 0.1% Triton X-100 for 5 min, and blocked with 1% BSA for 30 min. The cells were added to the primary antibody against Nestin/SOX2/CD133/Tuj1 or Texas Red conjugated phalloidin (Invitrogen, Waltham, MA, USA) for 2 h and FITC-conjugated second antibody. The samples were washed three additional times then mounted using Mowiol. The stained cells were viewed by a confocal microscope (TCS SP5, Leica, German). Antibodies and dilutions were as follows: rabbit polyclonal antibody Nestin (1:200) and SOX2 (1:100) both from Capital Bio Corporation in Beijing; mouse monoclonal CD133 (1:40, Miltenyi Biotec, Auburn, CA, USA); polyclonal antibody Tuj-1 (1:500, Life Technologies, Carlsbad, CA, USA).

### RT-PCR and qPCR

Total RNA was isolated from cells samples by Trizol Reagent. The quality and purity of RNA were assessed by the ratio of OD_260_ and OD_280_, which of the value of all samples ranged from 1.8 to 2.2. RNA integrity assessed the Agilent 2100 bioanalyzer (Agilent Technologies, Santa Clara, CA, USA). The RNA integrity number (RIN) value of all samples ranged from 8.1 to 8.9 (scale1–10), indicating high-quality RNA.

First-strand cDNA was prepared from total RNA (1 μg) by Prime Script™ RT reagent Kit With gDNA Eraser form RNA (Takara, Dalian China) according to the manufacturer’s specifications. One microliter of 5-fold dilution of cDNA and 0.4 μM of primer pair (Supplementary Table S1) were used in 20 μl reaction volume with SYBRs Premix Ex Taq II (Perfect Real Time; Takara, Dalian, China) in master cycler real plex (Eppendorf, Germany), 5 ng of template cDNA, 45 cycles: 95°C/15 s, 60°C/15 s. These qRT-PCR procedures were run in duplicate to correct for variances in loading. All PCR results were determined using the relative quantification method (2^−ΔΔ^Ct) with GADPH as the normalization control.

### Adipogenesis Differentiation

BM-MSCs (6 × 10^4^ cells/well) were seeded in 24-well plates and cultured at 37°C in a humidified atmosphere with 5% CO_2_ in DMEM supplemented with 10% volume fraction of FBS, 10 mmol/L dexamethasone, 10 mg/L insulin, 100 mg/L 1-methyl-3-isobutyl xanthine, 100 mg/L indomethacin, 100 U/ml penicillin, and 100 mg/L streptomycin. This adipogenesis differentiation medium was replaced every 3–4 days. After 14 days of culture, the cells can be processed for Oil Red O staining, to detect adipogenesis.

### Animal Model

Brain injury rat models were divided randomly into cell group (*n* = 20) and control group (*n* = 20; device as Supplementary Figure S2). Cell group rat tracer injected with CD-Dil tagged BM-NPCs 10 μl (1 million) through the microsyringe transplantation to cerebral injury rats, under the condition of the same set as control group with injecting medium. Movement function Wayne Clark test and grooming test were carried out respectively after transplantation of 1 day, 3 days, 7 days, and 30 days and 60 days. At the same time, 7 days, 30 days and 90 days after transplantation, brain tissue pathological conditions were detected, using immunofluorescence test to analyze Dil tagged BM-NPCs migration in brain injury and nerve cell markers NeuN. Antibodies and dilutions were as follows: neuron-specific enolase (NeuN) monoclonal, 1:200 (BD Bioscience).

### Histological Observations

After treatment, the brains were carefully excised, rinsed in PBS, and then fixed in 4% PFA. The samples were dehydrated in a graded ethanol series (70–100%) and embedded in paraffin. Five-micrometer sections were prepared. According to the standard procedures, samples were stained with hematoxylin and eosin (HE).

### Animal Behavior Test

Wayne Clark and grooming scores were carried out according to the score table (Bertelli and Mira, 1994; Finnie, 2001).

### Statistical Analysis

Data were expressed as mean ± SEM. Comparisons of mean values among the groups were compared using Student’s *t*-test. A five percent probability (*P* < 0.05) was used as the level of significance. Differences were considered statistically significant with *P* < 0.01.

## Results

### Characterization of BM-MSCs

**Figure 1:**
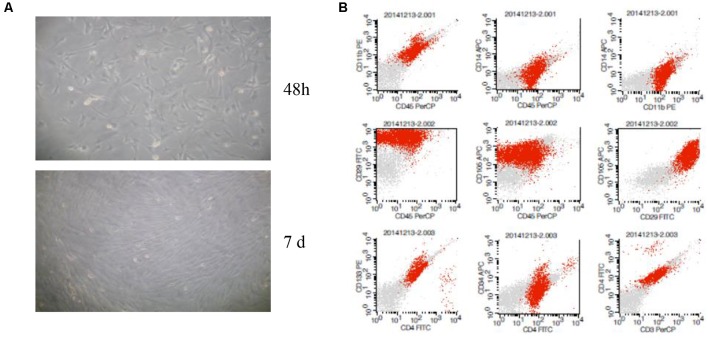
Characterization of bone marrow-derived mesenchymal stem cells (BM-MSCs). **(A)** Microscope morphological observation of BM-MSCs. **(B)** Analysis of BM-MSCs for the expression of surface markers by flow cytometric analysis.

BM-MSCs were generated using the whole bone marrow adherent culture method, identified by analysis extending cell morphology and the surface markers using flow cytometry (Figure 1A). The results showed that characteristic of BM-MSCs in accordance with the international appraisal standard of MSCs, identified by microscope morphological observation and the flow cytometry of CD34/45/3/4/11b/14/133 (−) and CD29/105 (+) (Figure 1B).

### Characterization of BM-NPCs

**Figure 2:**
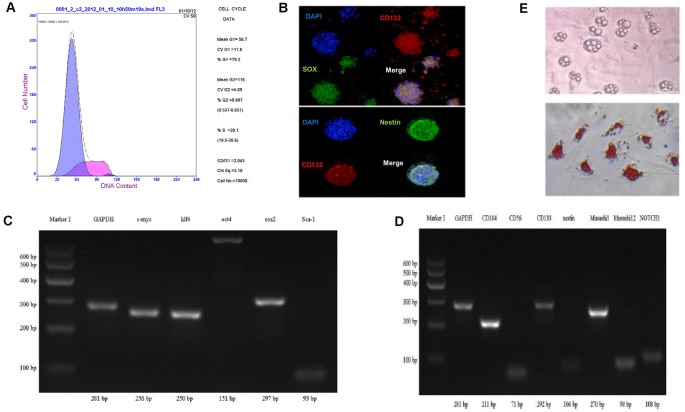
Characterization of bone marrow-derived neural progenitor cells. **(A)** The cell cycle by flow cytometry. **(B–D)** Identification of cell differentiation potential and neural progenitor cells related to gene protein expression using cell immunofluorescence staining and RT-PCR method. **(E)** Oil Red O staining to detect adipogenesis differentiation.

Through the method of neural stem cell suspension culture, bone marrow-derived sphere-like cells were harvested, which was measured by the flow cytometry cycle, and the results showed that 79.2% of the third generation of sphere-like cells was in G0/G1 phase (Figure 2A).

To analyze cell differentiation potential of sphere-like cells, cell immunofluorescence and RT-PCR method were carried out to detect the pluripotent surface markers expression. The results showed sphere-like cells express protein CD133, Sox2, and Nestin (Figure 2B).

To detect the mRNA level, semi-quantitative RT-PCR was executed. Pluripotent stem cell gene namely c-myc, klf4, sox2, Sca-1, oct4 and neural progenitor cells gene including Muashil1, CD184, CD133, CD56, Nestin, Muashil2, Notch1 were both stronger expressed in sphere-like cells (Figures 2C,D). To further confirm the pluripotency, sphere-like cells were induced to adipose differentiation. The result showed positive lipid drops with oil O staining (Figure 2E).

### BM-NPCs Differentiation Into Neuron-Like Cells

To analyze the differentiation ability of BM-NPCs to nerve cells, genetic level changes in the process of cell differentiation was detected using both direct adherent differentiation and neuron co-culture induction method.

**Figure 3:**
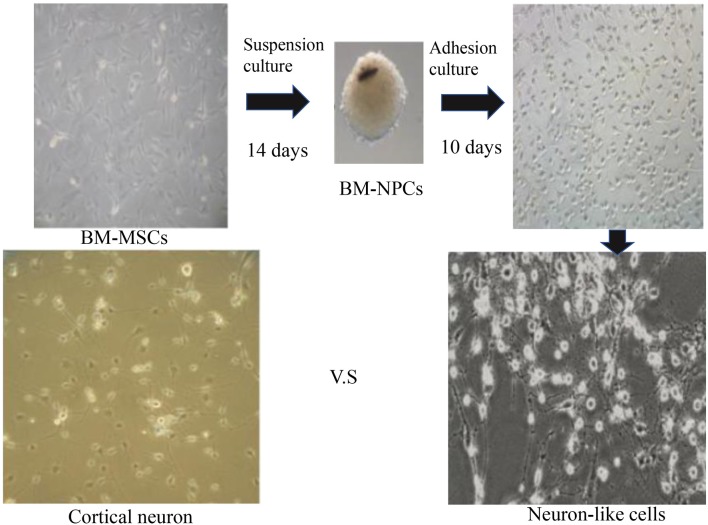
Direct adherent induces differentiation of bone marrow neural progenitor cells into neuron-like cells.

The third generation of BM-NPCs was cultured in neurons medium for 15 days. BM-NPCs adhered to the wall directly and neuron-like cells can be observed after 10 days, some of which like glial cells, linked with each other to grow. When continue to induce the other 5 days, a typical morphology of nerve cells, similar to normal cortex neuron cells and completely different from BM-MSCs, can be observed (Figure 3).

**Figure 4:**
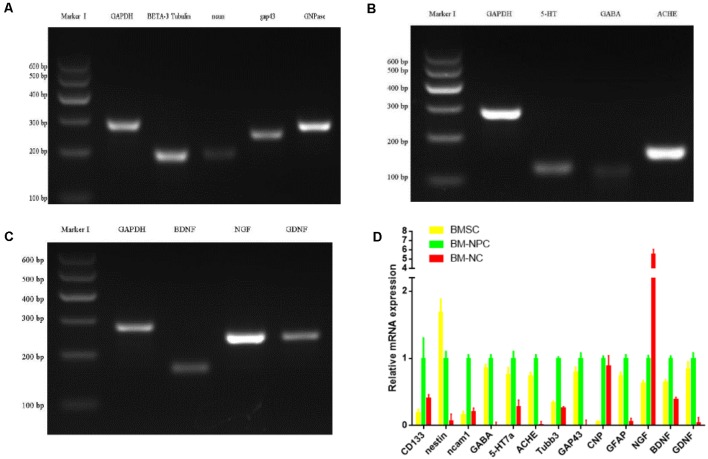
Direct adherent induces differentiation of bone marrow neural progenitor cells into neuron-like cells, whose neuronal markers were detected by semi-quantitative RT-PCR (**A–C** from BM-NC) and quantitative qPCR **(D)**.

**Figure 5:**
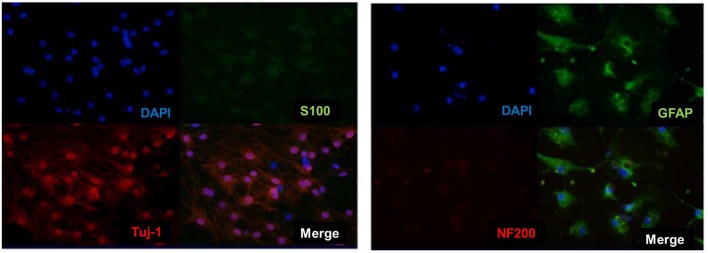
Direct adherent induced differentiation of bone marrow neural progenitor cells into neuron-like cells, whose neuronal markers Tuj-1 (+)/NF200 (−) and S100 (+)/GFAP (+) was detected by immunofluorescence.

Then analysis of neuronal markers was detected. Using semi-quantitative RT-PCR and quantitative qPCR to detect mRNA expression level before and after inducing BM-NPCs about neural stem cell marker genes (Nestin/CD56/CD133), the nerve cells marker genes (the beta-III-tubulin/Neun/5-HT/ACHE/GABA, and CNPase and neurotrophic factor gene NGF/BDNF/GDNF gene (Figures 4A–C). Quantitative gene expression results showed BM-NPCs was higher expression of neural progenitor cell gene CD56 and CD133 compared to BM-MSCs. What’s more, results showed higher CNPase expression and NGF nutrition factor gene-level increased significantly (Figure 4D). To confirm the expression of neuronal markers. Tuj-1/NF200 and glial cell marker GFAP were detected by immunofluorescence technique. These nerve-like cells expressed Tuj-1 (+)/NF200 (−) and GFAP (+)/S100 (+) (Figure 5).

**Figure 6:**
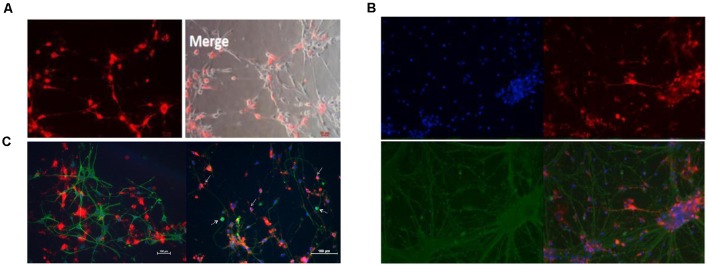
Neuron co-culture induced differentiation of bone marrow neural progenitor cells into neuron-like cells followed by immunofluorescence observations. **(A)** BM-NPCs cocultured with cortical neurons for 10 days. **(B)** BM-NPCs cocultured with cortical neurons for 15 days. **(C)** BM-MSCs (left) and BM-NPCs cocultured with the cortical neuron. CM-Dil (red), Tuj1 (green) and DAPI (blue).

In addition, the CM-Dil cells tracer tagged BM-NPCs was cocultured with the original generation cortex neurons for 10 days, the result showed neurons-like morphology and network growth with neurons (Figure 6A), like primary cortex neurons (Supplementary Figure S1). For 15 days, using an inverted microscope and immunofluorescence observations to detect neuronal markers Tuj-1 expression. When CM-Dil cells tracer tagged BM-NPCs in the more suitable environment for neuronal growth was co-culture with the original generation of cortex neuron cell, BM-NPCs can be differentiated into more typical neuron morphological characteristics, with Tuj1 fluorescent protein-positive expression, and normal neural network connected into the growth cells (Figure 6B). However, unlike BM-NPCs, BM-MSCs cocultured with neurons did not show the appearance (Figure 6C).

### Long-Term Survival of Bone Marrow-Derived Neurons Involved in Nerve Regeneration

Bone marrow-derived neural progenitor cells (BM-NPCs) transplantation in the brain injury rats was carried out followed by a search for the evidence about long-term survival *in vivo*, and participating in nerve regeneration of brain damage.

**Figure 7:**
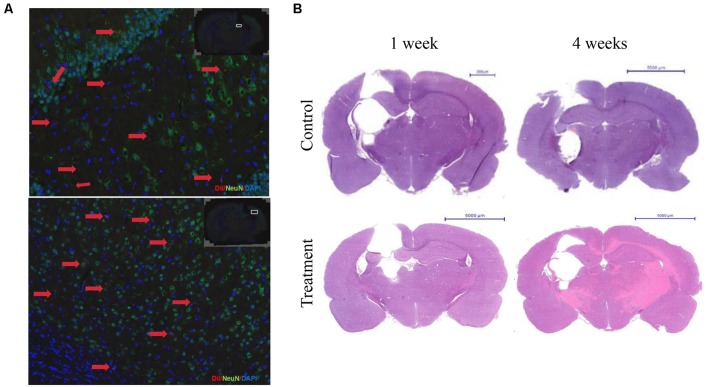
Bone marrow-derived neurons can long-term survival in brain injury rats and promote nerve regeneration. **(A)** The immunofluorescence staining results showed that the Dil labeled cells after 4 weeks of transplantation. Dil (red), NeuN(green), DAPI (blue). **(B)** HE staining after 1 or 4 weeks of transplantation.

First, the immunofluorescence results showed that 7 days of transplantation, the Dil labeled cells transplanted into the area of injury around brain tissue. However, Dil labeled cells still not show NeuN positive. Transplantation for 30 days, brain damage tissue around GFAP positive astrocytes, some Dil+ cells, in the region of the hippocampus and cerebral cortex neurons, with normal nerve cells express integration expressed NeuN. After 4 weeks, cell group was still visible Dil tagged positive cells expressed NeuN, which integrated in normal nerve cells in the brain tissue, and the damage zone of the surrounding tissue growth (Figure 7A).

Second, HE staining showed 1 week after cell transplantation, the control group damage surrounding tissue with edema, oven visible cystic cavity, significantly reduced the number of nerve cells, inflammatory cells infiltration around, cell group edema, lighter, cystic cavity range limit, glial cells. Transplantation for 4 weeks, focal brain injury recovered from the surrounding tissue, compared with the control group. The cystic cavity was small in a cell group, and the row of the surrounding cell of the class was neat, tissue edema and inflammatory cells disappeared (Figure 7B).

**Figure 8:**
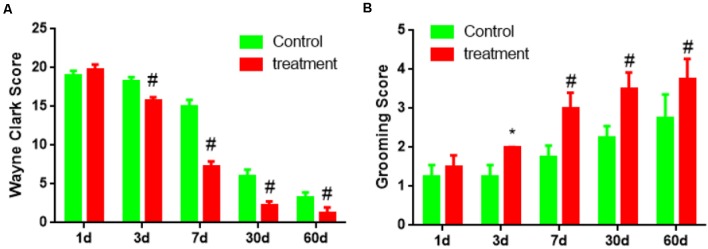
The behavioral score of brain damage rats after BM-NPCs transplantation. **(A)** Wayne Clark and **(B)** grooming score after transplantation for different time points. *P < 0.05, ^#^*P* < 0.01.

Third, the behavioral score showed two groups of Wayne Clark and grooming score results were no significant difference (*P* < 0.05) on 1 day. But transplantation for 3 days, 7 days, 30 days, 60 days, Wayne Clark, grooming score results had significant difference (*P* < 0.05 or *P* < 0.01). The cell group had better functional recovery (Figures 8A,B).

## Discussion

More and more focus on the capacity of stem cell-derived neural progenitor cells following brain injury (Pati et al., 2016), but less evidence ensure that these cells transdifferentiate into neural cells (Shinoyama et al., 2013). This article explored that bone marrow sphere-like cells were obtained from the BM-MSCs using the method of neural stem cell suspension culture, identified as BM-NPCs by neural progenitor cells gene and protein expression, and neural progenitor cells characteristic and nerve cell differentiation *in vitro*. Moreover, BM-NPCs transplantation improved the behavior and histological indexes of brain injury rats.

First, the pluripotent stem cell gene and neural progenitor cells gene were both stronger expressed in sphere-like cells, that suggested the bone marrow-derived sphere-like cells was pluripotent and able to differentiate into neural cells, in other words, they were bone marrow-derived neural progenitor cells (BM-NPCs; Figures 2A–E).

These cells adhered to the wall directly, then showed a typical morphology of neuron-like cells and nerve cells, different from BM-MSCs (Figure 3). Further, after induced differentiation of BM-NPCs, the Nestin, NCAM1, and CD133 gene expression decreased obviously because maybe the cells differentiate into different nerve cells. beta-III-tubulin/Neun/5-HT/ACHE gene expression also decreased obviously, which suggested that the “stick-to-wall” differentiation environment was not conducive to neuron differentiation, so these cells were unable to form mature neurons with neurotransmitter expression. Cells may easily differentiate into glial cells with cultivation over a long time because glial cells were more easy to survive and proliferate, then with higher CNPase and NGF nutrition factor expression (Figures 4A–D). What’s more, expression of neuronal markers suggested the more appropriate neuron growth environment, BM-NPCs might have the capacity to differentiate into mature functional neurons.

These results *in vitro* showed that suspension-cultured BM-NPCs have more ability to differentiate into nerve cells compared with BM-MSCs and that BM-NPCs induced neuronal cells were similar to fully mature neurons cell, however, a certain difference in the gene expression still exists. BM-NPCs probably have the ability to differentiate into functional neural cells in the appropriate environment, playing a role in the central nervous system injury disease (Figures 5, 6).

Finally, the results that BM-NPCs transplantation can promote brain injury of limb motor function recovery in rats, that supply the evidence of long-term survival in intracranial, integrating into damage brain and participating in nerve regeneration (Figures 7, 8). The behavioral score of brain damage rats improved 3 days after BM-NPCs transplantation. This time point was in accord with the study that concludes that the critical time period for manipulating endogenous NPCs following a spinal cord injury in rats was between 24 h when Nestin expression in ependymal cells increased and 1 week when astrocytes were activated in large numbers (Mao et al., 2016).

## Conclusion

This study identified the BM-NPCs and provided valuable experimental basis data for BM-NPCs transplantation, suggesting the alternative treatment of central nervous injury disease.

## Data Availability Statement

All datasets generated for this study are included in the article/Supplementary Material.

## Ethics Statement

The animal study was reviewed and approved by The ethics committee of Guangdong Provincial People’s Hospital.

## Author Contributions

WB wrote the manuscript. YZ revised the manuscript. MZ proposed the idea for the manuscript. WX created the figures. XL was responsible for checking the manuscript. WL, ML, and FY contributed to the analysis and interpretation of data. WB and YZ were responsible for data collection, study design, and critical review. Finally, we thank all patients who took part in this study.

## Supplementary Material

The Supplementary Material for this article can be found online at: https://www.frontiersin.org/articles/10.3389/fncel.2020.00049/full#supplementary-material.

## Conflict of Interest

The authors declare that the research was conducted in the absence of any commercial or financial relationships that could be construed as a potential conflict of interest.

## Footnotes

^1^https://clinicaltrials.gov/
