# Supplementary material for: Isolation and Characterization of Neural Progenitor Cells From Bone Marrow in Cell Replacement Therapy of Brain Injury
Source: Front Cell Neurosci. 2020 Mar 12;14:49. doi: 10.3389/fncel.2020.00049 (PMC7080866; doi:10.3389/fncel.2020.00049)
Supplement: Supplementary file 1 [file Table_1.DOCX]

**SUPPLEMENTARY INFORMATION**

## Table S1. The PCR primer sequences and expected PCR productive size were as follows:

| Gene Name | Primer^a^ | Productive size (bp) | |
| --- | --- | --- | --- |
| Rat c-myc | F GAGTCAGGGTCATCCCCATCA | | 256 |
|  | R CCAAGACGTTGTGTGTCCGC | |  |
| Rat Klf4 | F TCGGTCATCAGTGTTAGCAAAGG | | 250 |
|  | R CGGGACTCAGTGTAGGGGTAGT | |  |
| Rat OCT4 | F GTGTTCAGCCAGACAACCATCT | | 151 |
|  | R GCACCAGGGTCTCCGATTTG | |  |
| Rat Sox2 | F CGGAAAACCAAGACGCTCAT | | 297 |
|  | R GCTGGTCATGGAGTTGTACTGC | |  |
| Rat Sca-1 | F GAAAGCCGAAACTCTTCATCAT | | 93 |
|  | R ATGCCATATCATCGTCAGTTCC | |  |
| Rat CD184 | F GTGGGCAATGGGTTGGTAAT | | 211 |
|  | R CACTGCTGTAAAGGTTGACGGT | |  |
| Rat CD56 | F CAAGTCCCTAGACTGGAACGC | | 71 |
|  | R CCTTGGATTTTCCTTGCTGGT | |  |
| Rat CD133 | F AACCCAAAGTTACTCCTGTTCTCG | | 292 |
|  | R TTGAGTTCCCTGTCCACTGATG | |  |
| Rat nestin | F GGACTCAGAACAAGTGAATGGG | | 106 |
|  | R CTGTCCCTGTAATAGGAGTTCTTG | |  |
| Rat musashi-1 | F GGGTTTCGGCTTCGTCACTT | | 270 |
|  | R CCTCTGTGCCTGTTGGTGGTT | |  |
| Rat musashi-2 | F CTTCGGTTTCGTCACCTTCG | | 98 |
|  | R CAACTTTGGGGTCAATCGTCT | |  |
| Rat NOTCH1 | F TGTCATCTCCGACTTCATCTATC | | 108 |
|  | R GCAGCATCTGAACGAGAGTATC | |  |
| Rat beta-3 Tubulin | F GCATCTCCGAGCAGTTTACG | | 190 |
|  | R TCCTCGTCGTCATCTTCATACAT | |  |
| Rat NeuN | F CTTCCAGGGTCGTGTATCAGG | | 190 |
|  | R GTTCCGATGCTGTAGGTTGC | |  |
| Rat GAP43 | F GGAGATGGCTCTGCTACTACCG | | 239 |
|  | R GGCACATCGGCTTGTTTAGG | |  |
| Rat CNPase | F CCACTTTACTTTGGCTGGTTCC | | 264 |
|  | R CACCACATCCTGTTGGGCAT | |  |
| Rat 5-HT | F ATAGCTGATATGCTGCTGGGTT | | 123 |
|  | R AAAGAGCACATCCAGGTAAATCC | |  |
| Rat GABA | F CCATTGTCCTCTTCTCCCTCTC | | 121 |
|  | R TTTGCCTCCACTTCTACAGACC | |  |
| Rat ACHE | F CACCGTGCCTCCACATTGACT | | 166 |
|  | R CTGTGCGGGCAAAATTGGTC | |  |
| Rat BDNF | F TTGATGAGACCGGGTTCCCT | | 255 |
|  | R GTCCGTGGACGTTTGCTTCTT | |  |
| Rat NGF | F CATCACTGTGGACCCCAAACTGT | | 247 |
|  | R GTCCGTGGCTGTGGTCTTATCTC | |  |
| Rat GDNF | F AATGTCACTGACTTGGGTTTGG | | 245 |
|  | R CGTTTAGCGGAATGCTTTCTTA | |  |
| Rat GAPDH | F TTCCTACCCCCAATGTATCCG | | 281 |
|  | R CATGAGGTCCACCACCCTGTT | |  |

^a^ indicates sequence of forward (F) and reverse (R) primers.
