# Supplementary material for: Isolation and Characterization of Neural Progenitor Cells From Bone Marrow in Cell Replacement Therapy of Brain Injury
Source: Front Cell Neurosci. 2020 Mar 12;14:49. doi: 10.3389/fncel.2020.00049 (PMC7080866; doi:10.3389/fncel.2020.00049)
Supplement: Supplementary file 2 [file Presentation_1.pptx]

## Slide 1
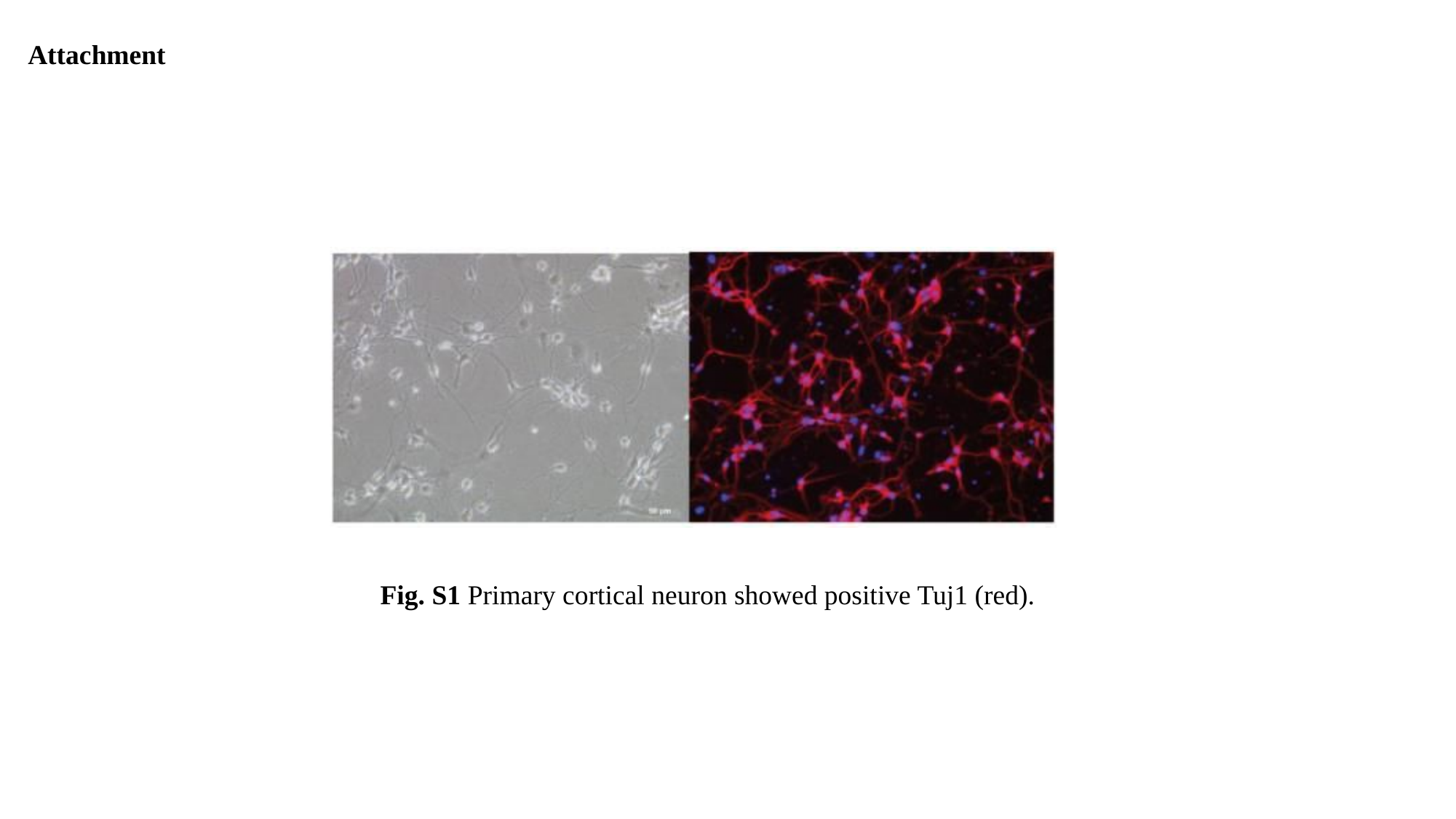

Attachment
Fig. S1 Primary cortical neuron showed positive Tuj1 (red).

## Slide 2
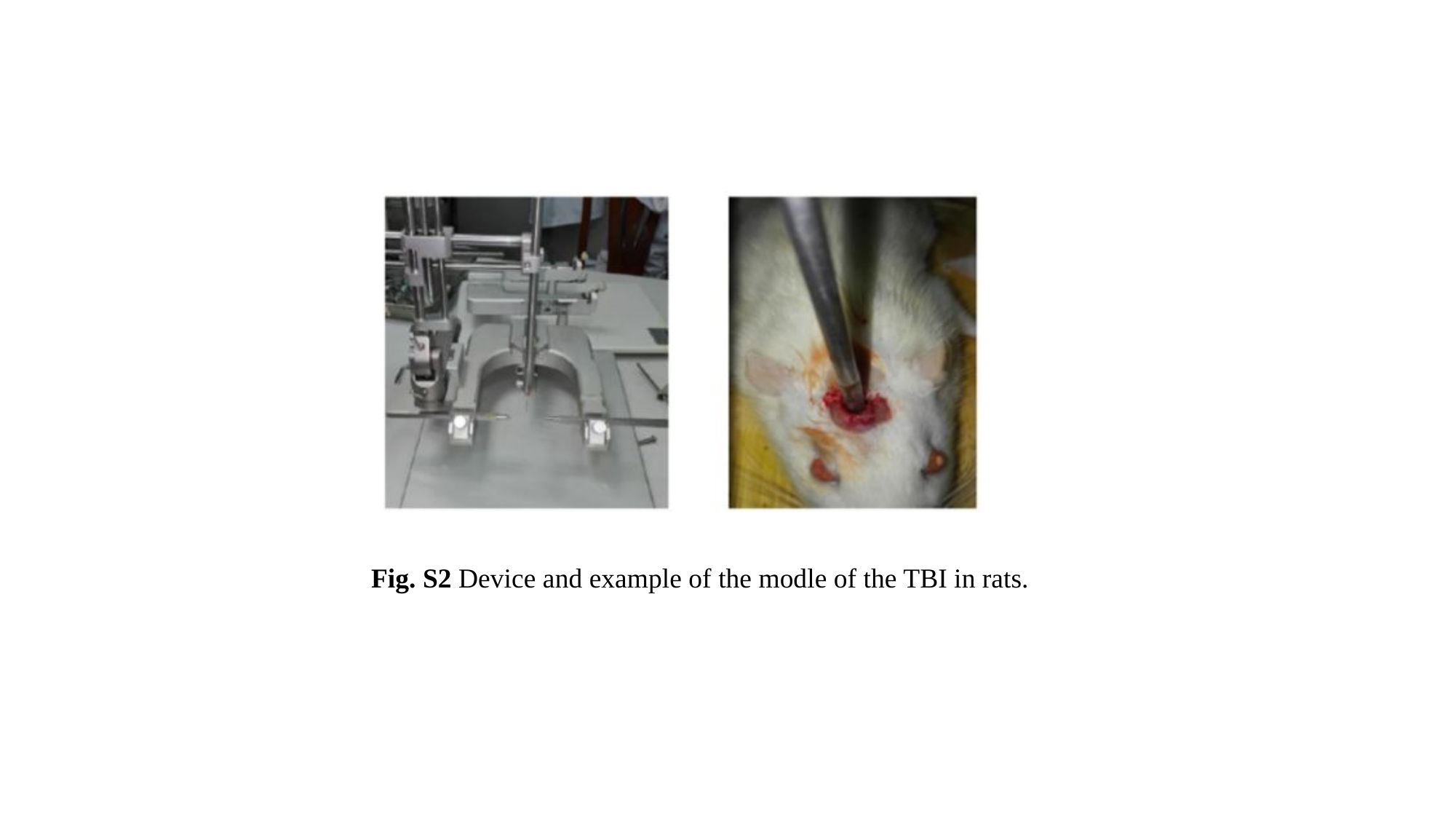

Fig. S2 Device and example of the modle of the TBI in rats.
